# Sex- and Diet-Specific Changes of Imprinted Gene Expression and DNA Methylation in Mouse Placenta under a High-Fat Diet

**DOI:** 10.1371/journal.pone.0014398

**Published:** 2010-12-21

**Authors:** Catherine Gallou-Kabani, Anne Gabory, Jörg Tost, Mohsen Karimi, Sylvain Mayeur, Jean Lesage, Elsa Boudadi, Marie-Sylvie Gross, Julien Taurelle, Alexandre Vigé, Christophe Breton, Brigitte Reusens, Claude Remacle, Didier Vieau, Tomas J. Ekström, Jean-Philippe Jais, Claudine Junien

**Affiliations:** 1 Inserm, AP-HP, Université Paris-Descartes, Faculté de Médecine, Hôpital Necker-Enfants Malades, U781, Paris, France; 2 INRA, UMR1198, UMR INRA/ENV Maisons-Alfort/CNRS: Biologie du Développement et Reproduction, (ENV Maisons-Alfort; CNRS), Physiologie Animale et Systèmes d'Elevage, Centre de recherche de Jouy-en-Josas, Jouy-en-Josas, France; 3 Laboratoire d'Epigénétique, CEA - Institut de Génomique, Centre National de Génotypage, Evry, France; 4 Laboratory for Medical Epigenetics, Department of Clinical Neuroscience, Centre for Molecular Medicine, Karolinska Institutet, Stockholm, Sweden; 5 Unité Environnement Périnatal et Croissance, EA 4489, Université des Sciences et Technologies de Lille, Villeneuve d'Ascq, France; 6 Laboratory of Cell Biology, Institute of Life Sciences, Catholic University of Louvain, Louvain-la-Neuve, Belgium; 7 SBIM, Université Paris Descartes, Paris, France; City of Hope National Medical Center, United States of America

## Abstract

**Background:**

Changes in imprinted gene dosage in the placenta may compromise the prenatal control of nutritional resources. Indeed monoallelic behaviour and sensitivity to changes in regional epigenetic state render imprinted genes both vulnerable and adaptable.

**Methods and Findings:**

We investigated whether a high-fat diet (HFD) during pregnancy modified the expression of imprinted genes and local and global DNA methylation patterns in the placenta. Pregnant mice were fed a HFD or a control diet (CD) during the first 15 days of gestation. We compared gene expression patterns in total placenta homogenates, for male and female offspring, by the RT-qPCR analysis of 20 imprinted genes. Sexual dimorphism and sensitivity to diet were observed for nine genes from four clusters on chromosomes 6, 7, 12 and 17. As assessed by *in situ* hybridization, these changes were not due to variation in the proportions of the placental layers. Bisulphite-sequencing analysis of 30 CpGs within the differentially methylated region (DMR) of the chromosome 17 cluster revealed sex- and diet-specific differential methylation of individual CpGs in two conspicuous subregions. Bioinformatic analysis suggested that these differentially methylated CpGs might lie within recognition elements or binding sites for transcription factors or factors involved in chromatin remodelling. Placental global DNA methylation, as assessed by the LUMA technique, was also sexually dimorphic on the CD, with lower methylation levels in male than in female placentae. The HFD led to global DNA hypomethylation only in female placenta. Bisulphite pyrosequencing showed that neither B1 nor LINE repetitive elements could account for these differences in DNA methylation.

**Conclusions:**

A HFD during gestation triggers sex-specific epigenetic alterations within CpG and throughout the genome, together with the deregulation of clusters of imprinted genes important in the control of many cellular, metabolic and physiological functions potentially involved in adaptation and/or evolution. These findings highlight the importance of studying both sexes in epidemiological protocols and dietary interventions.

## Introduction

There is no doubt that much of the increase in obesity can be attributed to lifestyle factors, such as the excess consumption of energy-rich foods, a decline in physical activity, inherited genetic and other factors [1]. However the ‘Developmental Origins of Adult Health and Disease’ (DOHaD) hypothesis provides an alternative hypothesis [2]. Maternal nutrient deprivation has been well characterised in this context. However little is known about the potentially deleterious effects of overnutrition, such as a typical hypercaloric Western diet rich in energy, saturated fats and sugar or a high-fat diet, on the health of offspring, potentially resulting in a metabolic syndrome phenotype in the offspring [3], [4], [5]. Obese and diabetic women are less fertile than women of normal weight, tend to consume more calories, particularly from fat [6], and have a higher rate of adverse pregnancy outcomes [7] and a higher risk of impaired breastfeeding [8]. The proportion of women of child-bearing age who are overweight (25%, 30%, and 50% in France, Germany, and the US, respectively) and do not eat an appropriate diet is significant and increases. We therefore need to identify ways of providing advice, evidence-based dietary recommendations, clinical treatments and counselling for these women and their babies.

There is increasing evidence to suggest that the placenta is involved in determining the risk of cardiovascular disease and cancer (reviewed in [9], [10], [11], [12], [13], [14 Thornburg, 2010 #6165]). The placenta is the primary means of communication and nutrient delivery to the foetus and is presumably involved in foetal homeostasis. It is therefore an appropriate organ for studies investigating how differences in maternal food consumption are sensed by the developing offspring [15]. In mice, the mature placenta (E14.5) consists of three principal layers: an outer layer of trophoblast giant cells, a middle spongiotrophoblast layer (sometimes called the junctional zone) and the innermost labyrinth [16], [17], [18]. Placental function follows a carefully orchestrated developmental cascade during gestation. Both the development and ongoing functions of the placenta may be dynamically regulated by environmental factors, including nutrient status and tissue oxygenation [19]. The timing of certain adverse incidents during development may therefore have a critical effect on the subsequent vasculature of the placenta or on trophoblast and placental function and foetal programming [20], [21].

Despite the important role of the placental in supplying nutrients to the foetus, very few studies have investigated the effects of general nutritional status on blastocyst development and implantation, subsequent placental development and the role of the placenta in adaptive epigenetic processes in response to nutritional stimuli [18], [22], [23], [24], [25], [26], [27], [28], [29], [30], [31], [32], [33]. Only two groups have explored the impact on the global placental gene response of changes in maternal diet [12], [34], [35]. The data obtained suggest that placental development is highly adaptable and that there are many possible types of compensation for suboptimal nutrition [36].

The placenta has long been considered to be an asexual organ, with most placental studies consistently pooling data for male and female placentae into a single group [37]. However, predisposition to metabolic disease differs between the sexes, with women more likely to develop obesity and men, cardiovascular disease. This sexual dimorphism may already exist during development. Indeed, there is mounting evidence to suggest that the sex of the embryo, through the embryo-derived tissues of the placenta, plays a significant role in determining foetal size, nutrition, morbidity and survival [37], [38]. Such differences may appear early, even before the gonads have developed, highlighting the important role of the sex chromosomes [39], [40] (reviewed in [41]). A molecular investigation of the extent to which male and female conceptuses react to the same maternal diet is therefore of interest. Only a handful of studies have reported differences between the sexes, in terms of the expression of individual genes or pathways, in male and female human and rodent placentae. These studies also addressed the impact of differences in the quality of the maternal diet on placental gene expression, with a systematic investigation of the relationship between diet and the expression of sexually dimorphic genes, providing insight into the different sensitivities of male and female foetuses to what the mother eats [12], [35], [37], [42], [43], [44], [45], [46].

Genomic imprinting is an epigenetic phenomenon in which specific mammalian genes are expressed preferentially from the allele inherited either from the father or from the mother. More than 80 genes are imprinted in humans and mice, and it is thought that there may be 100 to 500 imprinted genes in the entire genomes of these species [47], [48]. The placenta is notable amongst mammalian organs for its high and prolific expression of imprinted genes. [49], [50]. Most imprinted genes are grouped into clusters, each of which may contain a mixture of maternally and paternally expressed genes. These clusters are located at about 15 different sites on the chromosomes of the human genome, at sites syntenic to those in mice. These imprinted domains are co-ordinately regulated by imprinting centres, consisting of differentially methylated regions (DMRs) that are methylated either maternally during oogenesis or paternally during spermatogenesis. DMRs act via long-range mechanisms, such as antisense RNA interference, and through methylation-sensitive boundary elements (CTCF) [30], [51], [52], [53]. A network of imprinted genes, including *Zac1* and *H19*, which controls embryonic growth and may be the key to a common mechanism of gene regulation during mammalian evolution, has recently been described [54], [55]. Another important feature associated with imprinted gene loci is the presence of imprinted small non-coding RNAs clusters [56]. The complexity of imprinted domain regulation may also render these domains particularly susceptible to environmental changes of gene expression through nutrition during the prenatal period, beginning in the preimplantation embryo, and in the postnatal period [25], [57], [58], [59]. It has recently been shown that changes in postnatal growth induced by a maternal low protein diet (LPD) at the time of conception may be result partly from the sex-specific programming of imprinted gene expression within the preimplantation embryo itself [25]. The expression profile for imprinted genes has been shown to be altered in placentae from rat foetuses presenting IUGR [60], from infants with a low birth weight [61], [62]} or after superovulation in the midgestation placenta in human pregnancies [63]. Imprinted genes are dosage-sensitive. They encode proteins involved in common pathways and play multiple roles in the placenta, including regulation of the growth and transport capacity, thereby controlling the supply of nutrients to the foetus [53], [64], [65]. They may also directly regulate the growth rate of foetal tissues, thereby controlling foetal nutrient demand. Moreover genetic and molecular studies of the development and evolution of sexual dimorphism [66] have shown that epigenetic marks at imprinted gene DMRs are established in a sex-specific manner in bovine blastocysts, after somatic cloning [67]. It remains unclear how epigenetic modifications fix the effects of early environmental events, in a sex-specific manner, ensuring sustained responses to transient stimuli and resulting in modified gene expression patterns and phenotypes later in life [68].

There is convincing experimental evidence to suggest that epigenetic marks act as a memory of exposure to inappropriate environments in early life. These marks induce long-term changes in gene expression, potentially leading to disease in later life. Disturbed placental epigenetics has been demonstrated in cases of intrauterine growth retardation and small for gestational age, and also appears to be involved in the pathogenesis of pre-eclampsia and gestational trophoblastic disease (reviewed in [69]). Our aims are to identify how and where epigenetic modifications fix the effects of early environmental events (overnutrition associated with a deleterious uterine environment) to ensure sustained responses to transient stimuli, leading to the modification of gene expression patterns and phenotypes later in life [41], [68]. We investigated the ways in which maternal diet might influence imprinted gene expression and epigenetic DNA methylation at the whole-genome level and in imprinted gene DMRs in male and female foetuses during the last third of pregnancy, when morphological development of the placenta is complete [70]. We investigated the impact of a high-fat diet (HFD) during pregnancy on the expression of 20 imprinted genes. We observed sex- and diet-specific differential expression of imprinted genes from four clusters, in the placentae of E15.5 male and female offspring from the litters of pregnant mice fed a HFD since the first day of gestation. Global DNA methylation also showed sex- and diet-specific differences.

## Results

### Developmental studies

Pregnant females were weighed on the day of the vaginal plug and at E15.5. Overall, HFD-fed mothers had a weight gain that was higher than CD fed mothers (14.0±0.3g vs 10.3±0.3g, p = 0.0001), but when the weight gain is reported in relation to the number of fœtuses, there was no difference in weight gain between animals on the two different diets (2.0±0.13g vs 1.8±0.13g, p = 0.2173). We determined placental and foetal weights at E15.5 for 110 female and 81 male foetuses from mothers on the CD, and for 99 female and 103 male foetuses from mothers on the HFD (Table 1). No gross physical deformities were observed in either the control or the high-fat group and litter size was not affected by the HFD. The main effect of sex was on placental weight, the placenta being heavier for male than for female foetuses, regardless of diet (*p*<0.0001): 9.7% heavier for the CD and 11.2% heavier for the HFD. Diet affected placental weight regardless of the sex of the offspring (*p*<0.0001), with the HFD resulting in a 6.4% heavier placenta for females and a 7.9% heavier placenta for males. Embryo weight did not differ significantly between the sexes and was unaffected by diet. Remarkably, the foetal weight to placental weight ratio index (FPI), reflecting nutrient transfer from the placenta to the foetuses, was affected by diet (*p* = 0.0039), the HFD reducing the FPI, and was different according to the sex (*p*<0.0001), females having a greater FPI than males.

**Table 1:** Foetal and placental weights in the high-fat diet mouse model.

	Female on CD(n = 110)	Female on HFD (n = 81)	Male on CD(n = 99)	Male on HFD (n = 103)	*p*, effect of diet	*p*, effect of sex	*p*, interaction diet*sex
**Foetal weight (g)**	0.439±0.006	0.448±0.006	0.453±0.007	0.454±0.005	NS	NS	NS
**Placental weight (g)**	0.116±0.002	0.123±0.002	0.127±0.002	0.137±0.002	<0.0001	<0.0001	NS
**Foetus/Placenta ratio**	3.846±0.058	3.714±0.074	3.629±0.070	3.378±0.062	0.0039	<0.0001	NS

Values are expressed as means ± SEM and statistical significance is expressed as a *p* value from two-way ANOVA with post-hoc testing. NS: Not significant.

### Analysis of gene expression by RT-qPCR: effects of sex and of diet

We used RT-qPCR to analyse the expression in the placenta of 20 genes located in seven imprinted clusters on five chromosomes. We studied six pools of female foetuses and six pools of male foetuses from mothers fed the CD, and seven pools of female foetuses and seven pools of male foetuses from mothers fed the HFD. Each pool comprised all the placentae from male or female mice from the same litter (n = 3–7).

ANOVA indicated a main effect of sex on the expression of three genes (*Peg10*, *Slc22a1*, *Slc22a2;* Table 2), males having a weaker expression than females. Post hoc analysis (comparing two groups) showed that the expression of four genes (*Peg10, Asb4, Peg3* and *Slc22a2*) was significantly weaker in male offspring than in female offspring and that the expression of *Ascl2* was significantly weaker in female offspring than in male offspring when the mother was fed the CD (Table 2).

**Table 2:** Placental mRNA levels for 17 candidate genes, determined by RT-qPCR, in the high-fat diet mouse model.

Gene	Chromosome	Allele expression	Placenta-specificimprint	Female on CD(n = 6 pools)	Female on HFD(n = 7 pools)	Male on CD(n = 6 pools)	Male on HFD(n = 7 pools)	*p*, effect of diet	*p*, effect of sex
***Gatm***	**2**	**M**	**+**	**0.210±0.053**	**0.138±0.021**	**0.149±0.059**	**0.116±0.028**	**NS**	**NS**
***Sgce***	**6**	**P**		**59.675±5.563**	**56.960±3.065**	**50.619±5.186**	**59.216±7.426**	**NS**	**NS**
***Peg10***	**6**	**P**		**1.214±0.071** 1	**1.172±0.092**	**0.892±0.118** 1	**1.005±0.124**	**NS**	**0.0296**
***Ppp1r9a***	**6**	**M**	**+**	**1.220±0.112**	**1.171±0.112**	**0.922±0.096**	**1.088±0.144**	**NS**	**NS**
***Pon3***	**6**	**M**	**+**	**0.458±0.087**	**0.517±0.101**	**0.613±0.110**	**0.419±0.045**	**NS**	**NS**
***Pon2***	**6**	**M**	**+**	**1.039±0.146**	**1.063±0.116**	**0.910±0.114**	**0.960±0.150**	**NS**	**NS**
***Asb4***	**6**	**M**		**10.480±0.932** 2	**8.315±1.042**	**7.629±0.639** 2	**8.795±0.931**	**NS**	**NS**
***Peg3***	**7**	**P**		**2.806±0.549** 3	**1.822±0.281**	**1.579±0.097** 3	**1.885±0.294**	**NS**	**NS**
***Igf2P0***	**7**	**P**		**10.534±1.664**	**7.389±1.002**	**8.133±2.103**	**8.354±1.329**	**NS**	**NS**
***Igf2***	**7**	**P**		**13.26±1.05**	**12.70±0.76**	**12.31±0.81**	**11.87±1.09**	**NS**	**NS**
***H19***	**7**	**M**		**11.82±0.57**	**11.19±0.56**	**11.07±1.16**	**10.93±0.73**	**NS**	**NS**
***Ascl2***	**7**	**M**	**+**	**6.8±0.35** 9	**10.56±1.57**	**11.57±0.62** 9	**11.32±1.73**	**NS**	**NS**
***Dlk1***	**12**	**P**		**0.857±0.150** 8	**0.449±0.062** 8	**0.496±0.124**	**0.360±0.055**	**NS**	**NS**
***Gtl2/Meg3***	**12**	**M**		**33.750±4.553**	**30.796±4.770**	**26.952±5.139**	**32.124±6.670**	**NS**	**NS**
***Rtl1***	**12**	**P**		**1.564±0.353**	**0.952±0.142**	**1.140±0.236**	**0.811±0.095**	**0.0405**	**NS**
***Dio3***	**12**	**P**		***2.385*** **±** ***0.342*** 4	***1.576*** **±** ***0.176*** 4	**1.641±0.536**	**1.224±0.147**	**NS**	**NS**
***Igf2r***	**17**	**M**		**0.701±0.104**	**0.542±0.075**	**0.523±0.113**	**0.430±0.020**	**NS**	**NS**
***Airn***	**17**	**P**		**8.622±0.830**	**10.030±1.454**	**11.665±1.108**	**10.513±1.375**	**NS**	**NS**
***Slc22a1***	**17**	**M/P**		**0.042±0.006** 5	**0.028±0.003** 5	**0.029±0.003**	**0.022±0.002**	**0.0100**	**0.0146**
***Slc22a2***	**17**	**M**	**+**	**0.528±0.078** 6	**0.970±0.248**	**0.209±0.076^6.7^**	**0.573±0.086** 7	**0.0182**	**0.0333**
***Slc22a3***	**17**	**M**	**+**	**0.620±0.127**	**0.368±0.066**	**0.485±0.099**	**0.296±0.024**	**0.0148**	**NS**

The 18S RNA was used as an endogenous control, to normalise the amount of template for each gene. Values are expressed as means ± SEM and statistical significance is expressed as a *p* value following two-way ANOVA with post-hoc testing. Superscripts indicate significant differences between groups with the same superscript in post-hoc tests:

1 : 0.0413;

2 : 0.0303,

3 : 0.0426,

4 : 0.0499,

5 : 0.0421,

6 : 0.0179,

7 : 0.0130,

8 : 0.0222,

9 : 0.0001.

NS: Not significant.

ANOVA indicated a main effect of diet on the expression of three genes located within the same cluster on chromosome 17 (*Slc22a1*, *Slc22a2, Slc22a3*) and one gene on chromosome 12*: Rtl1*. *Slc22a2* expression was increased by the HFD, particularly in males, whereas expression levels for *Slc22a1* and *Slc22a3* were lower when the mother was fed the HFD. For *Igf2r,* we observed a non-significant trend towards lower levels of expression when the mother was fed the HFD (Table 2). No effect of diet was detected for the expression of 16 transcripts (*Gatm, Sgce, Peg10, Ppp1r9a, Pon3, Pon2, Asb4, Peg3, Igf2P0, Igf2, H19, Ascl2, Gtl2, Dlk1, Dio3, and Airn*). No interaction between sex and diet was detected for any of the genes analysed (Table 2).

Post-hoc analysis was carried out to check for a sexually dimorphic response to diet in the two groups. *Dlk1* and *Dio3,* showed no global (ANOVA) differences, but the HFD significantly decreased the expression of these genes in females only.

**Figure 1:**
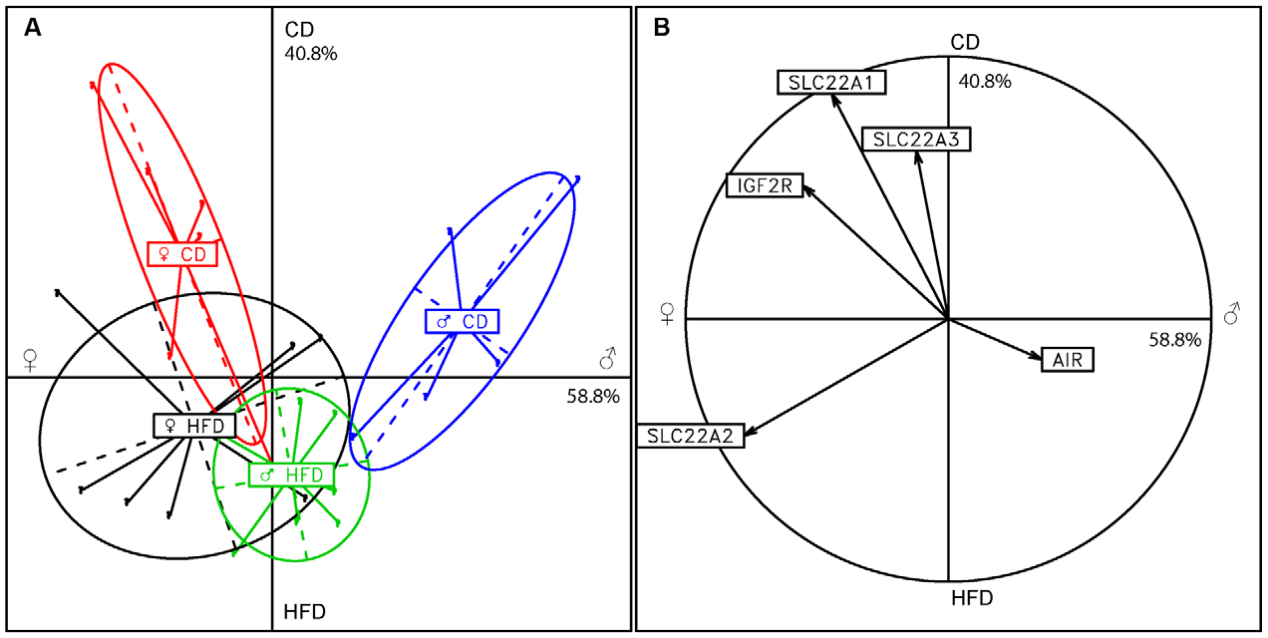
Linear discriminant supervised clustering analysis. The five genes mapping to chromosome 17 for which RT-qPCR was carried out (*Slc22a1, Slc22a2, Slc22a3, Igf2r, Air*) were studied by a linear discriminant approach. (A) Pooled results from individuals were projected onto the first two discriminant axes, which accounted for 99.6% of the inter-class variability. Inertia ellipses represent intra-class variability for each group. Discrimination power increases with the compactness and distance of the ellipses. (B) The correlation circle indicates the contribution of the five genes to the discriminant axes.

We used another statistical approach, supervised clustering analysis, to confirm the discriminant value of the cluster of genes on chromosome 17. The five genes for which RT-qPCR was carried out that mapped to chromosome 17 (*Slc22a1, Slc22a2, Slc22a3, Igf2r, Airn*) were studied with a linear discriminant approach (Figure 1). Pooled samples from individuals were projected on the first two discriminant axes, which accounted for 99.6% of the inter-class variability (58.8% and 40.8%, respectively). The compactness and distance of the inertia ellipses confirm that the expression profiles for these five genes can be used to discriminate between pool samples as a function of sex and diet, although this discrimination seems to be better for animals in the CD group than for those in the HFD group (Fig. 1A). As shown by the correlation circle, *Slc22a2* contributes mostly to sex discrimination, being overexpressed in females and in the presence of a HFD. By contrast, *Slc22a1, Slc22a3* and *Igf2r* contribute mostly to diet discrimination and are overexpressed in females and in the presence of the CD. By contrast, *Airn* makes little contribution to the discrimination axes (Fig. 1B).

### Analysis of gene expression by *in situ* hybridization

**Figure 2:**
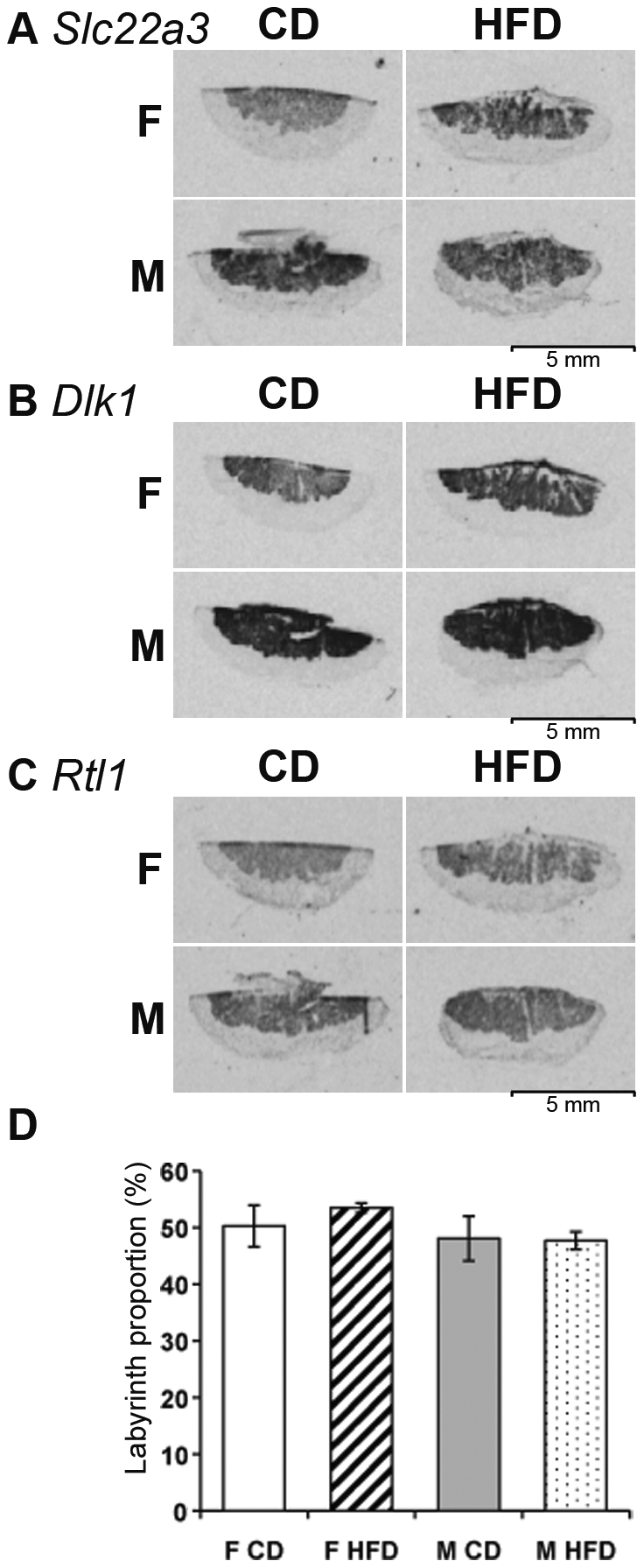
Analysis of gene expression by *in situ* hybridization. Detection by *in situ* hybridisation of *Slc22a3* (A), *Dlk1* (B) and *Rtl1* (C) in of the placentae of female (F) or male (M) mouse embryos from mothers fed a control (CD) or high fat (HFD) diet. No difference in the distribution of these RNAs was observed, all of which were restricted to the labyrinth layer. The histograms (D) show the proportion of the labyrinth, this area being similar between the four groups.

The location of the mRNAs for *Dio3, Rtl1, Dlk1, Slc22a1, Slc22a2*, and *Slc22a3* was determined by *in situ* hybridisation, in four samples per group. The expression of *Slc22a1*, *Slc22a2* and *Dio3* was too weak for detection by this technique. In male and female placentae from animals fed the CD, the expression of *Slc22a3*, *Dlk1* and *Rtl1* was detected in the labyrinth zone, as previously reported (Figure 2). No difference between males and females was found in the location of the signals in CD and HFD samples. We investigated possible variation in the proportions of the placental layers, using ISH images for *Rtl1* to measure the zone of expression (i.e. the labyrinth zone) and to compare the area obtained with the total area of the placenta, for the four different placentae of each group analysed. We detected no differences (Figure 2D). These data strongly suggest that the variation in expression of these imprinted genes is not due to differences in the relative size of the labyrinth zone with respect to the other layers [71], [72].

### Sexual dimorphism in methylation of the DMR of the *Igf2r* cluster in HFD mice

**Figure 3:**
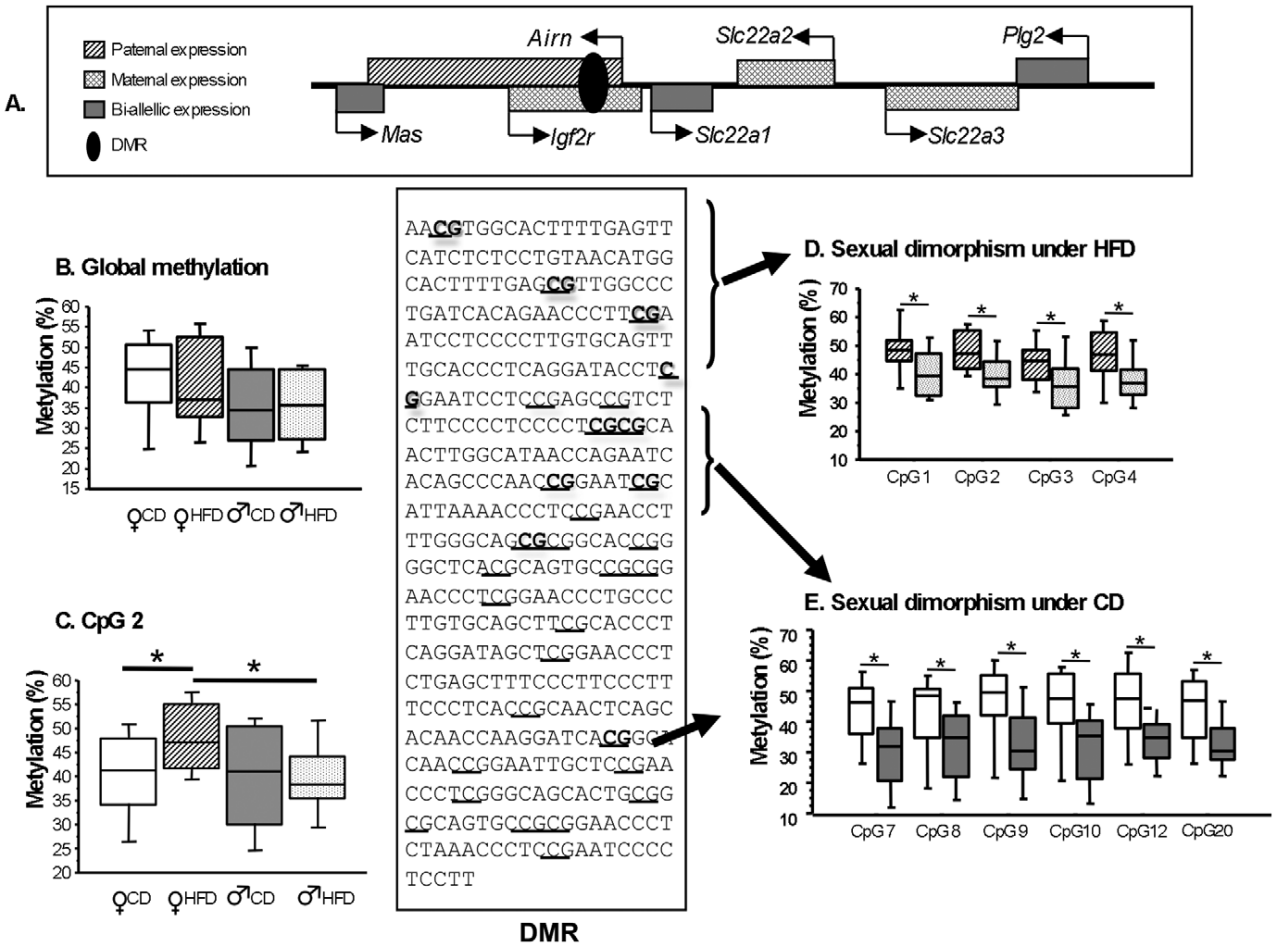
Schematic diagram of the *Igf2r* region on mouse chromosome 17. (A), Box plot representing global methylation (B), specific CpG2 (C), sexual dimorphism under HFD (D), and sexual dimorphism under CD (E) obtained by bisulphite sequencing analysis of the DMR *Igf2r* encompassing 30 CpGs. The 30 CpGs are underlined. CpGs displaying sex-specific methylation under HFD and CD are shown in bold. Differences between methylation profiles were analysed by Mann-Whitney tests (*p*<0.05). F =  female, M =  male.

The RT-qPCR analysis detected two clusters (*Dio3, Rtl1, Dlk1* on chromosome 12 and *Slc22a1, Slc22a2*, *Slc22a3* on chromosome 17) displaying modified expression of genes involved in regulation of homeostasis. We investigated the epigenetic mechanisms potentially responsible for the changes in expression of these genes, by exploring DMR methylation. Based on the results of the supervised clustering analysis, showing greater discrimination with the *Igf2r* cluster, we decided to analyse the methylation levels of the DMR of this cluster. We used the bisulphite-sequencing method to analyse a 490 bp fragment encompassing 30 CpG in the DMR within intron 2 of the *Igf2r* gene [73] (Figure 3A). We studied DNA from the placentae of 12 female and 9 male foetuses from mothers fed the CD and 14 female and 15 male foetuses from mothers fed the HFD, corresponding to three CD litters and four HFD litters. We analysed 22 to 40 clones for each individual placenta, to ensure that we had enough sequences for the reliable determination of % of methylation for each CpG.

The analysis of all 30 CpG, as a group, revealed no significant difference between the sexes or between diets (Figure 3B). By contrast, when we analysed each CpG separately, we found significant differences in methylation between the sexes and between the diets. Four clustered CpGs showed statistically significant differences in DNA methylation when the group of females placentae under the HFD were compared to the group of male placentae under a HFD (numbers 1, 2, 3, 4 with 17%, 16%, 17%, 16%, respectively) (Figure 3D). For CpG2, only females showed a difference in methylation under the HFD (Figure 3C). Five clustered CpGs (number 7, 8, 9, 10, 12 with 18%, 12%, 19%, 17%, 19%, respectively) and CpG20 (16%) showed statistically significant differences in DNA methylation when the group of females placentae under a CD were compared to the group of male placentae under a CD (Figure 3E).

We checked for the presence of binding sites/responsive elements for chromatin remodelling factors and transcription factors (Genomatix®) in the *Igf2r* DMR (File S1). We identified potential consensus binding sites for more than 15 different factors in the region encompassing CpGs 1 to 4, which were differentially methylated as a function of diet. Binding sites or responsive elements for the following transcription factors/chromatin remodelling factors are compatible with the *Igf2r* DMR, from nucleotides 4 to 129: Pax4, Smarca3, Vbp, Pax6, Yy1, Oct1, Nrf2/Arp, Ppar/Rxr, Egr3, Rxr, Mzf1, Sry/Sox9, Gcm1, Stat6, Nudr/Deaf-1. Several of the corresponding genes, present in the *Ensembl* database, have been shown to display significant levels of expression in the placenta (*Pax4, Smarca3, Nrf2/Arp, Ppar/Rxr, Egr3, Rxr, Stat6*), consistent with a potential role for these factors.

For the *Dlk1, Rtl1, Dio3* and *Gtl2* cluster, we used the more rapid and quantitative pyrosequencing approach, which had already been optimised in our laboratory. We identified statistically significant differences in methylation for two CpGs, at positions 1 and 8 in the DMR, with an effect of sex (CpG 1 and 8; *p* = 0.044 and *p* = 0.045) and an effect of diet (CpG 1; *p* = 0.034). However, in contrast to the Igf2r DMR, these differences were not large enough (less than 10%) for us to be able to speculate about their role (data not shown).

### Sexual dimorphism in global DNA methylation in mouse tissues and placenta

**Figure 4:**
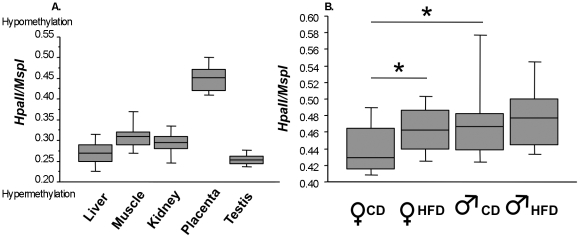
Box plot representing global methylation of the genome. Results obtained by LUMA, in five different tissues from six-month-old mice on the CD (liver, muscle, kidney, testis and placenta) at E15.5 (A), and from the placentae of female and male foetuses from mothers fed the CD or the HFD (B). Differences between methylation profiles were analysed by Mann-Whitney tests (*p*<0.05). Completely unmethylated DNA would have an *Hpa*II*/Msp*I ratio of 1.0, whereas 100% methylated DNA would have an *Hpa*II*/Msp*I ratio close to zero [121].

Global DNA methylation was assessed by the LUMA technique, in which the ratio of genomic DNA digested by the methylation-sensitive enzyme *Hpa*II to that digested with the methylation-insensitive enzyme *MspI* indicates the level of cytosine demethylation. Figure 4A shows the distribution of relative methylation in five different tissues of six-month-old female mice fed the CD. The tissues studied were the liver (females n = 41), skeletal muscle (gastrocnemius) (females n = 10), kidney (females n = 20), and testis (males n = 39), together with the placenta at E15.5 (15 males and 25 females, n = 40). As previously reported, we found that the level of global DNA methylation in the placenta was markedly different from that in other somatic tissues (*p*<0.001) [74], [75], [76]. Differences between tissues were also observed in the % methylation, except between the liver and testis and between the muscle and kidney (Figure 4A). An effect of sex was observed under the CD. Male placentae displayed lower levels (3.3%) of methylation than female placentae (*p* = 0.035). Diet had an effect on global % methylation, but only in females (2.4% *p* = 0.032). Female placentae from mothers fed the HFD displayed lower levels of methylation (Figure 4B). The levels of methylation of SINE/B1 and LINE1, common repetitive elements, were assessed by bisulphite-pyrosequencing. No differences were observed for the entire collection of sequences explored (data not shown), demonstrating that the difference in global methylation observed was not due to these repetitive sequences [77].

## Discussion

It has been suggested that changes in imprinted gene dosage in the placentae may compromise the prenatal control of nutritional resources [78]. However, the underlying mechanisms remain unclear. This study is the first to demonstrate that the placentae of male and female foetuses from mothers fed a HFD display changes in both the expression of selected imprinted genes from different clusters, and in genome-wide and CpG-specific DNA methylation, with these changes differing between the sexes.

Experimental and epidemiological studies in humans and animal models have demonstrated that predisposition to impaired glucose tolerance, blood pressure and coronary heart disease, are associated with either low or high FPI [9], [10], [11], [12], [13], [14], [23], [79], [80], [81]. We show here that FPI is sexually dimorphic. Female mice have a higher FPI than male mice. Moreover, we show, for a large number of animals (n = 81 to 110 per group), that feeding the mother a HFD for the first 15 days of gestation only is sufficient to increase placental weight significantly, for both males and females. However, this treatment had no effect on fetal weight, for the foetuses of either sex. Consequently, FPI was also affected by diet, with the HFD reducing FPI in both males and females. It would therefore be interesting to investigate the changes in the respective size of the placenta and foetus at term following the administration of a HFD to pregnant mice [82]. Indeed the FPI changes should not be overestimated since we are looking at E15.5, not at term as in published human data. Altogether the impaired nutrient transfer from the placenta to the foetus, as reflected by the sex- and diet-specific alterations of the FPI, is consistent with the role of placenta *in utero* in sexual dimorphic programming and subsequent impaired responses in adulthood [81].

### Sexual dimorphism for imprinted genes

Only a few studies have reported the differential expression of individual gene products in human male and female placentae [42], [43], [44], [45]. Five of the 20 genes analysed in this study displayed sexual dimorphism when the mother was fed the CD: *Peg10, Asb4, Peg3, Slc22a2, Ascl2*. This proportion of sexually dimorphic genes is similar to that reported for other tissues or developmental stages [40], [41].

In a similar study performed at E12.5, in mice fed a low-fat diet, a very high-fat diet or an intermediate chow diet, Mao *et al.* observed that female placentae displayed more striking changes in gene expression with diet than male placentae [12]. This greater reactivity of females was also observed in total embryonic cells taken from mice at E10.5, before sexual differentiation [39]. Remarkably, these cells responded differently to the applied dietary stressors, even before the production of foetal sex hormones. In our study, carried out at E15.5, six genes (*Slc22a1*, *Slc22a2, Slc22a3, Rtl1, Dlk1* and *Dio3*) displayed changes in expression pattern when the mother was fed the HFD. We observed sex-specific sensitivity to the HFD, with effects limited to or more pronounced in the female placenta for *Dlk1*, *Dio3, Slc22a1* or to the male placenta for *Slc22a2* only. Our results are therefore consistent with previous findings that female placentae display more striking changes in gene expression in response to maternal diet than male placentae. As suggested by Penaloza *et al*., this difference in cell behaviour and sensitivity appears to be driven by the genetic sex of the cells from the outset, with the effects of factors such as hormones subsequently being superimposed on this difference [39]. Concerning the chromosome 17 cluster, (*Slc22a2, Slc22a1 and Slc22a3*), the sex steroid hormone oestrogen down-regulates renal organic cation transport in animals and may contribute to sex-related differences in xenobiotic accumulation and excretion [83], [84], [85]. However, caution is required when extrapolating transport-related sex differences between species and organs. These data on sexual dimorphism in organic cation transport are nonetheless potentially interesting when trying to understand the differences between the sexes in terms of the response in the placenta. For the chromosome 12 cluster, an effect of diet was observed for the paternally expressed *Dlk1, Rtl1 and Dio*3 genes, but not for the maternally expressed *Gtl2/meg3* genes, with female placentae again more sensitive than male placentae to the effects of the HFD.

One potential limitation of our study is that the placenta contains mixed cell populations. Changes in the proportions of the different cell populations could generate the differences observed, independently of changes in DNA methylation or gene expression in a single cell population [86]. Most expression studies in mice have analysed whole placentae [12], [34], [87], [88], but this analysis should ideally be based on an isolated cell population, making it possible to draw direct conclusions [89], [90]. We addressed this concern, by performing *in situ* hybridisation to visualise the expression of six genes that were differentially expressed and belonged to two clusters of dysregulated imprinted genes, on chromosomes 12 and 17. We detected no difference in the location of the signals or in the size of the zone of expression, for *Rtl1, Dlk1* and *Slc22a3,* between the male and female offspring of mice fed the CD and HFD. We cannot exclude the possibility of ectopic expression below the level of detection of this technique, but our data strongly suggest that the variation in expression of these genes, which are principally expressed in the labyrinth zone, is not due to a gross enlargement or reduction of the labyrinth zone with respect to the other layers.

### Function of the dysregulated imprinted genes

The body has various broad-specificity transporters for the elimination of environmental toxins and metabolic waste products. The non-neuronal monoamine transporters are polyspecific organic cation transporters (*Oct1, 2,* and *3* or *Slc22a1, 2,* and *3*). They control signal transmission by removing released transmitters, such as dopamine, noradrenaline, adrenaline, 5-hydroxytryptamine and histamine, from the extracellular space [91], [92]. Monoamine concentrations are normally kept low in the placenta. In humans, pre-eclampsia, which is characterized by high blood pressure and proteinuria, is one of the most common causes of perinatal and maternal morbidity and mortality. Monoamine transporters may protect against this condition by preventing vasoconstriction in the placental vascular bed, thereby ensuring stable blood flow to the foetus. In pre-eclampsia, hyperactivity of the sympathetic nervous system and high levels of circulating vasoactive substances, such as monoamines, have been observed. *SLC22A3* expression levels have been shown to be significantly lower in pre-eclamptic placentae than in normal placentae [93]. Defective expression of the genes encoding these monoamine transporters might account for the high concentrations of monoamines in patients with pre-eclampsia. The low level of expression of *Slc22a3* reported here may have led to similar disturbances in vasoconstriction and nutrient transport, accounting for the lower FPI.

The *Dio3, Rtl1, Dlk1* genes of the other cluster were expressed less strongly under a HFD than under a control diet. Interestingly, the maternally expressed *Gtl2* gene was not affected, precluding the involvment of this gene in the changes in gene expression of the paternally expressed *Dio3, Rtl1,* and *Dlk1* genes [94]. The *Rtl1* gene (retrotransposon-like 1), plays a determinant role in the foeto-maternal interface of mouse placenta [95]. *Rtl1* is essential for the maintenance of foetal capillaries, and both its loss and its overproduction cause late-foetal or neonatal death in mice [72]. There is evidence to suggest that genomic imprinting and gene expression at the *Dlk1/Dio3* imprinted domain may play a role in controlling adipocyte proliferation and differentiation [96], [97]. However, the roles of *Dio3* and *Dlk1* in placenta remain unknown. Thus, the decrease in the expression of *Slc22a3* and *Rtl1* under a HFD may contribute to changes in vascular function, resulting in the misregulation of nutrient transfer to the foetus. This led us to explore the DMR of these two clusters.

### Differential CpG methylation of the *Igf2r* DMR

The transporter genes *Slc22a2* and *Slc22a3* are imprinted specifically in mouse placenta [32]. The promoters of the repressed paternal alleles of these genes do not display DNA methylation [98]. The *Igf2r* imprint control element (ICE), which is a DMR containing 30 CpG, plays a crucial role in regulating many imprinted genes in this cluster. We therefore investigated whether adaptation of the nutrient supply to foetal demand in pregnant mice fed a HFD involved the ICE/DMR regulating these important placental transporter systems. We decided to investigate methylation of the DMR, despite previous reports that the difference in methylation is associated with regulation of monoallelic expression, rather than with expression levels *per se*.

In an analysis of all 30 CpG of the ICE together, we found no statistically significant difference between the sexes or the two diets. However, we observed sex- and diet-specific differential methylation of individual CpGs within two subregions of the DMR. Significantly different levels of methylation between the sexes were found for the first four CpGs in foetuses from mothers fed the HFD. Similarly, different levels of methylation between the sexes were found for the next five CpG and for CpG 20, in foetuses from mothers fed the CD. CpG 2 was the only CpG displaying both dietary and sexual dimorphism.

Bioinformatic analysis suggested that the CpGs displaying sex- and diet-specific differential methylation in the DMR might lie within recognition elements or binding sites for transcription factors or factors involved in chromatin remodelling, or within a higher-order chromatin architecture: Pax4, Smarca3, Nrf2/Arp, Ppar/Rxr, Egr3, Rxr, Stat6. PPAR-alpha and -gamma agonists increase *Slc22a1* gene transcription, thereby increasing the levels of the corresponding protein and increasing cellular organic cation uptake [99]. These data suggest that PPAR/RXR is one of the most likely candidate transcription factors, as the HFD contains well-known lipid ligands for these nuclear receptors. It is also tempting to speculate that the factors binding to this subregion may also interact with each other in a fatty acid-controlled transcriptional process. The helicase-like transcription factor (HLTF/SMARCA3) belongs to the SWI/SNF family of proteins. These proteins remodel chromatin in various cellular processes. Another member of the SMARC (SWI/SNF-related matrix-associated actin-dependent regulator of chromatin) family has recently been shown to function as a coactivator of another member of the nuclear receptor family [100]. However, it must be kept in mind that high-fat also correlates with low carbohydrate in the present diet, thus potentially influencing pathways involved in growth such as the insulin- and related signaling pathways.

It remains unclear whether and how these altered methylation profiles directly affect the expression profiles of the imprinted genes *Slc22a2*, and *Slc22a3* and that of the non-imprinted *Slc22a1* gene. The silencing of the paternal allele of the three imprinted genes (*Igf2r, Slc22a2* and *Slc22a3*) requires the expression, in *cis,* of *Airn,* which overlaps with the promoter of one of these genes (*Igf2r*) [101]. We observed no change in *Airn* and *Igf2r* expression, a decrease in *Slc22a1* and *Slc22a3* expression and an increase in *Slc22a2* expression in the placenta in mice fed the HFD. [98]. Sex- and diet-specific changes in the methylation of groups of CpGs in the DMR may alter the influence of *Airn* RNA in different ways for the three genes (*Igf2r, Slc22a2 and Slc22a3*). The promoters of these genes display no differential DNA methylation, therefore, histone modifications, which are likely to underlie the regulation of placental imprinted genes, may constitute an avenue of investigation. However, we were unable to study such modifications due to the mode of placenta sampling in this study.

For the *Dlk1, Rtl1, Dio3* and *Gtl2* cluster, in contrast to the Igf2r DMR, the differences in DNA methylation were modest. However, as shown very recently by Kagami et al, there is an additional functional DMR in this cluster. The IG-DMR, the DMR studied in the present report, and the Gtl2-DMR function as imprinting control centers in the placenta and the body, respectively [102].

### Diet-induced changes in DNA methylation in placenta

The maternal HFD leads to a global hypomethylation in placenta compared to the maternal CD. This hypomethylation is statistically significant in females only. Could this hypomethylation be due to a reduction in methyl donor supply in the diet? To our knowledge, according to reports on food intake of high energy diets during gestation in rodents, the mothers either adjust their food intake in terms of calories regardless of the diet [103] or increase the caloric intake [104], [105]. In the case of the HFD and CD used in the present study (Research diets HFD D12492 and CD D12450B) the supply in vitamines is the same (40 kcal for 4057 kcal diet). Therefore, although the average daily food intake was not measured we can assume that the supply in vitamins B9 and B12 was not decreased in the HFD animals. Thus it is difficult to relate the hypomethylation observed to either an identical or increased level of vitamin supply. A global hypomethylation was also observed in brains of offspring of HF fed mice mothers [106]. However it remains difficult to speculate about the potential role of the highfat/low carbohydrate composition of the diet on the one-carbon metabolism, in the absence of relevant mechanisms to account for a potential link.

### Sexual dimorphism and global methylation

To our knowledge, this is the first report of sexual dimorphism for DNA methylation in the placenta under a control diet. This dimorphism may be due to the presence of an inactive X (Xi) chromosome in the female. In mouse extraembryonnic tissues, X chromosome inactivation is imprinted to occur selectively on the paternal X chromosome [107]. However, Weber *et al.* overturned previous views by showing that Xi was hypermethylated at only a subset of gene-rich regions and, unexpectedly, displayed overall hypomethylation with respect to its active counterpart [108]. Hellman *et al.* have shown that the active X (Xa) chromosome in females has levels of allele-specific methylation twice those of Xi. A bipartite methylation-demethylation program results in Xa-specific hypomethylation at gene promoters and hypermethylation at gene bodies in both male and female active Xa chromosomes [109].

We investigated this difference in methylation further, by assessing the methylation levels of the two major repetitive elements containing most of the genomic 5-methylcytosine bases: LINE-1 (long interspersed nucleotide element-1) and SINE-1 (short interspersed nucleotide element-1), represented by human Alu elements and the homologous mouse B1 elements. The methylation levels of both LINE-1 and SINE-1 have been reported to be a good indicator of cellular 5-methylcytosine level (i.e., global DNA methylation level) [110], [111]. In our placenta model, no difference in the level of LINE-1 or B1 repetitive element methylation was observed between the sexes or between the diets, CD and HFD. These differences are therefore probably located in non-genic regions, gene bodies and centromeric heterochromatin.

It has been suggested that the inherently lower level of methylation in the placenta than in other tissues [74], [75], [76] may render this organ highly susceptible to the effects of environmental factors, altering epigenetic patterns [32], [36], [112], [113], [114]. Consistent with this hypothesis, there have been several reports of global changes in DNA methylation in the placenta associated with IUGR, pre-eclampsia or undernutrition in the mother [36], [112], [113], [115]. Surprisingly, in this study, only females were sensitive to the HFD, resulting in undermethylation. These observations in mouse support the suggestion put forward by V. Clifton that sexually dimorphic differences in the growth and survival of the foetus are mediated by the sex-specific function of the human placenta [37], [38].

### Conclusion

Evidence in favour of non-genetic transgenerational inheritance is accumulating, in some cases with conspicuous, marked sexual dimorphism both for the mode of transmission and for the resulting effects [41]. Finely tuned aspects of the developmental programme, specific to one sex, may be more sensitive to specific environmental challenges, particularly during developmental programming and gametogenesis, but also throughout the individual's life, under the influence of sex steroid hormones. These findings highlight the importance of studying both sexes in epidemiological protocols or dietary interventions, both in humans and in experimental animal models. They pave the way to explorations concerning the possible targeting, by fatty acids and other nutrients, of conspicuous regions in the genome harbouring binding sites for the recruitment of diet- and tissue-specific chromatin remodelling complexes.

## Materials and Methods

### Ethics statement

All experiments on animals were conducted in accordance with the European Communities Council Directive of 1986 (86/609/EEC). Our laboratory has accreditation from the French Ministry of Agriculture for experimentation with mice (No. A 75-15-02). Approval of full details of the study by an ethics committee is not required under French laws.

### Experimental design and nutritional treatments

Four-week-old DBA/2 male and C57BL/6J female mice were obtained from Harlan® and housed in groups until mating. All animals were maintained under controlled light (12 h light/12 h dark cycle, light on at 07:00) and temperature (22±2°C) conditions.

The mice were allowed access to water and the control diet *ad libitum*. After two weeks of adaptation, DBA/2 male mice were mated with C57BL/6J female mice in the evening. The following day (day 0.5), if a vaginal plug was observed, females were fed either a HFD or a CD *ad libitum* for 15 days. The pregnant females were killed at E15.5, and placentas and foetuses were dried, weighed and frozen in liquid nitrogen before storage at −80°C. Diets were supplied in pellet form by Research Diets (New Brunswick, USA; CD: D12450B, HFD: D12492). For the CD, 10% of calories were in the form of fat, 20% were in the form of protein and 70% were in the form of carbohydrates. For the HFD, 60% of calories were in the form of fat, 20% were in the form of protein and 20% were in the form of carbohydrates [116].

DNA was extracted from the leg of mouse foetuses, using the DNeasy Tissue Kit (Qiagen). The sex of the foetus was determined by PCR of the SRY gene, as previously described [117].

### RNA extraction

Total RNA was extracted from rodent placenta with the RNeasy Mini kit® (Qiagen S.A., Courtaboeuf, France) and its concentration was determinated by measuring absorbance at 260 nm. RNA quality was assessed by agarose gel electrophoresis.

### Reverse transcription-quantitative PCR (RT-qPCR)

We determined mRNA levels for the genes of interest by reverse transcription followed by quantitative PCR (RT-qPCR). First-strand cDNAs were synthesised from 2(μg of total RNA in the presence of 50 ng random hexamers (GE Healthcare, Saclay, France), 400 nM dNTPs and 200 U Superscript™ II RNase H Reverse Transcriptase (Invitrogen, Cergy-Pontoise, France), according to the manufacturer's instructions. We checked that there was no DNA contamination by amplifying the 101 bp of the Ucp2 gene, using forward 5′-TGTCGAAGCCTACAAGAC-3′and reverse 5′-CAGCACAGTTGACAATGG-3′primers.

RT-qPCR analyses were carried out with the Absolute Blue qPCR SYBr Green Rox Mix (Thermo Scientific, Courtaboeuf, France), using an ABI PRISM 7300 apparatus, according to the manufacturer's instructions. Each reaction was carried out in a final volume of 25μl, in triplicate. Standard curves were generated for each run from 10-fold dilutions of cDNAs, to determine primer efficiency. Controls lacking reverse transcriptase were carried out alongside quantitative RT-qPCR for experimental samples, with SYBr Green. The controls consistently yielded no amplification below 40 cycles, using the above protocol. The 18S rRNA control was used to normalise the amount of template for each sample. Data were analysed with Microsoft Excel. The list of primers and real-time PCR assay conditions are available upon request.

### Bisulphite-cloning-sequencing methylation assay

DNA was isolated from mouse placentae using the DNeasy Tissue Kit (Qiagen). The isolated DNA was then treated with sodium bisulphite, using the EZ DNA Methylation Gold Kit (Proteigene, Saint-Marcel, France). The bisulphite-converted DNA was amplified by semi-nested PCR, using the primers *Igf2r* 13B-4, Igf2r 13B-2 and *Igf2r* 13B-5, as previously described [73]. The PCR products were purified with the Qiaquick PCR purification kit (Qiagen, Courtaboeuf, France), cloned with the PMOSblue Blunt Ended Cloning Kit (GE Healthcare, Saclay, France) and sequenced. The bisulphite treatment was more than 98% efficient. Quality control was carried out and methylation profiles were analysed with BiQ Analyzer software [118].

### Bisulphite quantitative pyrosequencing methylation assay

We treated 1 µg of genomic DNA with sodium bisulphite, using EpiTect® 96 bisulphite (Qiagen, Courtaboeuf, France) according to the manufacturer's instructions. Quantitative DNA methylation analysis of the bisulphite-treated DNA was performed by pyrosequencing or, in the case of several sequencing primers, by serial pyrosequencing [119]. Regions of interest were amplified from 25 ng of bisulphite-treated mouse genomic DNA, with 5 pmol of forward and reverse primers, one of which was biotinylated. Assays for the *Dlk1-Rtl-Dio3-Gtl2* cluster were performed as previously described [120].

DMR assays for the B1 repeat, as a surrogate for global DNA methylation changes, were performed as previously described [111] (Table 3). Primers for the LINE-1 element were designed to amplify nucleotides 64-326 of the consensus sequence (GenBank: D84391.1). Standard reaction conditions were HotStar Taq buffer, 1.6 mM MgCl_2_, 100 µM dNTPs and 2U HotStar Taq polymerase (Qiagen, Courtaboeuf, France) in a 25 µl volume. The PCR program consisted of 50 cycles of 30 s at 95°C, 30 s at the annealing temperature and 20 s at 72°C. Purification of the PCR product with streptavidin Sepharose HP beads (GE Healthcare, Uppsala, Sweden) and hybridization of the biotinylated PCR products and the sequencing primer were conducted as described in the PSQ96 sample preparation guide, using a vacuum filtration sample transfer device (Pyrosequencing AB, Uppsala, Sweden). Sequencing was performed on a PSQ 96MA system with the PyroGold SQA reagent kit, according to the manufacturer's instructions, and the results were analyzed with Q-CpG software V.1.0.9 (Pyrosequencing AB) [119].

**Table 3:** Bisulphite pyrosequencing analysis of repeated B1 and LINE sequences.

Regions	PCR primers	Productlength	Hybridization temperature (°C)	Sequencingprimers	CpGnumber	Sequencelength
***B1 repetitive elements***	**Forward**: GGTGGTGGTGGTGGTTGAGATAGReverse: AATAACACACACCTTTAATCCCAACACT	147	69	**Primer 1** TTTGTAGATTAGGTTGGTTT	3	45
***LINE1***	**Forward**: AGAATTTGATAGTTTTTGGAATAGG **Reverse**: ACTACCTCAATACCTCTATACTTCC	262	61	**Primer 1** GGTAGTATTTTGTGTGGGT	6	63
				**Primer 2** GATTTAAGTTATAGTAGTAG	5	35
				**Primer 3** TTGAATAGGTGAGAGGG	2	38

### Luminometric methylation assay (LUMA)

This assay was performed as previously described [121]. Briefly, genomic DNA (200 to 500 ng) was cleaved with *Hpa*II*+Eco*RI, and *Msp*I+*Eco*RI in two separate reactions in 96-well pyrosequencing plates. Digestion reactions were run in the PSQ96 MA system (Biotage AB). Peak heights were calculated with PSQ96 MA software. The *Hpa*II*/Eco*RI and *Msp*I*/Eco*RI ratios were calculated as (dGTP+dCTP)/dATP for the corresponding reactions. DNA methylation was assessed by calculating the *Hpa*II*/Msp*I ratio or, more precisely, by calculating the *(Hpa*II*/Eco*RI)/(*Msp*I*/Eco*RI) ratio.

### Statistical analysis

All data are expressed as means ± standard error (SEM). The effects of sex and diet on expression of the 18 genes tested were assessed by two-way ANOVA with post hoc testing (p<0.05), carried out with Statview (SAS Institute, Inc., Cary, NC). Supervised clustering analysis, using a linear discriminant approach, was performed with the ade4 package (http://pbil.univ-lyon1.fr/ADE-4) in the R statistical environment (http://www.r-project.org/). Differences between methylation profiles were analysed by carrying out Mann-Whitney tests with Statview.

### In situ hybridisation

Sections (12 µm thick) of some control and HFD placentae (n = 4) were mounted on gelatin-coated slides, dried and kept at −80°C. *In situ* hybridisation was performed as previously described [122]. The *Dlk1* probe was kindly provided by Dr. A. Ferguson-Smith (Cambridge, UK) and the *Slc22a3* probe was provided by Dr. D. Barlow (Vienna, Austria). *Dio3*, *Rtl1*, *Slc22a1* and *Slc22a2* probes were obtained by PCR on placental cDNA with the following primers: Dio3-F ATTCACCCTATGTCATCCCCCAGC and Dio3-R TCCTGAGAGCAAGCCAAAAACG at 54°C, Rtl1-F GCCCAGGAACACTATGTGGAACTC and Rtl1-R AAGTCTCATCATCTGCCTCCCTCG at 65°C, Slc22a1-F GAAGAGAACCACTCAAGCGGTAAGG and Slc22a2-R AGACAAGCGAGGGTCACATTCAAC at 54°C, Slc22a2-F AGACAGGTTTGGGCGGAAGTTC and AAGCAGAAGTTGGGCAGAGTCACG at 54°C. PCR fragments (489, 400, 435 and 315 bp, respectively) were inserted into pCR II vectors according to the TOPO TA cloning protocol (Invitrogen). The probes were linearised and labelled with [^35^S]-dUTP (1,300 Ci/mmol, Amersham Biosciences, Germany), with the Sp6/T7 Transcription Kit (Roche Diagnostics, Germany). Controls included hybridisation with a sense probe; no specific hybridisation signal was observed under these conditions, for any of the sense probes. For each probe, all the slides were placed against a single X-ray film (Biomax-MR, Kodak, France). All autoradiographs were digitised during the same session. For Slc22a3, Dlk1 and Rtl1, the signal was measured for 4 slides per placenta and 4 placentae per group. The results are expressed in OD x mm^2^. The measure of the proportion of the labyrinth corresponds to *Rtl1* signal surface to total surface ratio.

## Supporting Information

File S1 Search for potential transcription factor binding sites in the 490 bp of the DMR, including the 30 CpGs, with Genomatix.
(0.09 MB DOC)
