# Supplementary material for: Sex- and Diet-Specific Changes of Imprinted Gene Expression and DNA Methylation in Mouse Placenta under a High-Fat Diet
Source: PLoS One. 2010 Dec 21;5(12):e14398. doi: 10.1371/journal.pone.0014398 (PMC3006175; doi:10.1371/journal.pone.0014398)
Supplement: File S1 — Search for potential transcription factor binding sites in the 490 bp of the DMR, including the 30 CpGs, with Genomatix. (0.09 MB DOC) [file pone.0014398.s001.doc]

**Supplemental data**

| Family/MatrixName | **Optimized matrix threshold** | **Position**  **(from-to)** | **Strand** | **Core similarity** | **Matrix**  **similarity** | **Sequence**  **(italics: ci-value > 60**  **capitals: core sequence)** |
| --- | --- | --- | --- | --- | --- | --- |
| V$**PAX6/PAX4_PD.01**  PAX4 paired domain binding site | 0.91 | 4-22 | + | 1.000 | 0.928 | gtgG*CA*Ctttt*g*agttcat CpG1 |
| V$**RUSH/SMARCA3.02**  SWI/SNF-related, matrix-associated, actin-dependent regulator of chromatin, subfamily a, member 3 | 0.98 | 5-15 | + | 1.000 | 0.992 | tggc*ACTT*ttg |
| V$**PARF/VBP.01**  PAR-type chicken vitellogenin promoter-binding protein | 0.86 | 21-37 | + | 1.000 | 0.866 | atctctcctG*TAA*catg |
| V$PAX6/PAX6.02  PAX6 paired domain and homeodomain  are required for binding to this site | 0.87 | 22-40 | - | 0.864 | 0.872 | tgccatgttA*CAG*gaga*g*a |
| V$**YY1F/YY1.02**  Yin and Yang 1 repressor sites | 0.94 | 25-43 | - | 1.000 | 0.942 | aagtgC*CAT*g*t*tacaggag |
| V$OCT1/OCT1.05 Octamer-binding factor 1 | 0.89 | 32-46 | + | 1.000 | 0.899 | aa*CAT*Ggc*a*c*t*tttg |
| V$**RUSH/SMARCA3.02**  SWI/SNF-related, matrix-associated, actin-dependent regulator of chromatin, subfamily a, member 3 | 0.98 | 36-46 | + | 1.000 | 0.992 | tggc*ACTT*ttg |
| V$**NR2F/ARP1.01**  Apolipoprotein AI regulatory protein 1, NR2F2, DR1 sites | 0.82 | 39-63 | - | 0.809 | 0.883 | Tgatcag*gg*ccaac*G*C*TCa*aaagtg  **CpG2** |
| V$PERO/PPAR_RXR.02PPAR/RXR heterodimers, DR1 sites | 0.69 | 42-64 | - | 0.752 | 0.750 | Gtgatcag*ggccAACGctca*aaa  **CpG2** |
| V$EGRF/EGR3.01Early growth response gene 3 product | 0.77 | 44-60 | + | 1.000 | 0.773 | Ttga*GCGTtggcc*ctga CpG2 |
| V$RXRF/RXR_RXR.01Retinoid X receptor homodimer, DR1 sites | 0.78 | 54-78 | - | 0.888 | 0.815 | Attcgaagg**gttct**g**tG**A**TCa**gggc  **CpG3** |
| V$**MZF1/MZF1.02**  Myeloid zinc finger protein MZF1 | 0.99 | 77-87 | - | 1.000 | 0.990 | aa*GGGG*aggat |
| V$**SORY/SOX9.02**  SRY (sex-determining region Y) box 9 | 0.94 | 78-94 | - | 1.000 | 0.963 | actgcACAAg*g*ggagga |
| V$**GCMF/GCM1.01**  Glial cells missing homolog 1, chorion-specific transcription factor GCMa | 0.85 | 98-108 | + | 1.000 | 0.882 | ca*CCCTcag*ga |
| V$STAT/STAT6.01STAT6: signal transducer and activator of transcription 6 | 0.84 | 105-123 | + | 0.793 | 0.860 | Agga*TACC*tcg*gaa*tcctc CpG4 |
| V$DEAF/NUDR.01NUDR (nuclear DEAF-1-related transcriptional regulator protein) | 0.73 | 110-128 | + | 1.000 | 0.822 | Acc*TCGG*aat*cctccg*agc  **CpG4** **CpG5** |
| V$DEAF/NUDR.01NUDR (nuclear DEAF-1-related transcriptional regulator protein) | 0.73 | 111-129 | - | 1.000 | 0.887 | Ggc*TCGG*agg*attccg*agg CpG6 CpG5 CpG4 |
| V$EGRF/CKROX.01Collagen krox protein (zinc finger protein 67 - zfp67) | 0.88 | 132-148 | - | 1.000 | 0.900 | Cg*a*g*GGGAggg*gaagag CpG7 |
| V$**MZF1/MZF1.02**  Myeloid zinc finger protein MZF1 | 0.99 | 132-142 | - | 1.000 | 0.997 | ga*GGGG*aagag |
| V$**MAZF/MAZ.01**  Myc-associated zinc finger (MAZ) | 0.90 | 134-146 | - | 1.000 | 0.969 | aggg*GAGG*g*g*aag |
| V$SP1F/SP1.01Stimulating protein 1, ubiquitous zinc finger transcription factor | 0.88 | 134-148 | - | 0.807 | 0.900 | Cgag*GGGAggg*gaag CpG7 |
| V$**MZF1/MZF1.03**  Myeloid zinc finger protein MZF1 | 0.95 | 137-147 | - | 1.000 | 0.994 | ga*GGGGa*gggg |
| V$MAZF/MAZ.01Myc-associated zinc finger protein (MAZ) | 0.90 | 139-151 | - | 1.000 | 0.964 | Gcgc*GAGG*g*g*agg CpG7/8 |
| V$E2FF/E2F4_DP1.01E2F-4/DP-1 heterodimeric complex | 0.84 | 141-157 | + | 1.000 | 0.844 | Cc*c*ct*CGCG*ca*a*cttg CpG7/8 |
| V$**OCT1/OCT1.01**  Octamer-binding factor 1 | 0.77 | 150-164 | - | 1.000 | 0.828 | gt*TATGccaag*ttgc |
| V$**CP2F/CP2.02**  LBP-1c (leader-binding protein-1c), LSF (late SV40 factor), CP2,SEF (SAA3 enhancer factor) | 0.84 | 151-169 | - | 0.875 | 0.859 | tT*C*T*G*gttatgc*c*aagttg |
| V$**PARF/DBP.01**  Albumin D-box binding protein | 0.84 | 152-168 | - | 1.000 | 0.902 | tctgg*TTATg*ccaagtt |
| V$**SNAP/PSE.02**  Proximal sequence element (PSE) of RNA polymerase III-transcribed genes | 0.73 | 154-172 | + | 0.892 | 0.772 | c*t*t*g*g*C*A*TAa*ccag*aa*tca |
| V$**GFI1/GFI1.02**  Growth factor independence 1 | 0.90 | 165-179 | + | 1.000 | 0.929 | cag*AATC*acagccca |
| V$ETSF/ELK1.02Elk-1 | 0.91 | 175-195 | + | 1.000 | 0.971 | Gcccaacc*GGAA*tcgcattaa  **CpG9 CpG10** |
| V$CART/CART1.01Cart-1 (cartilage homeoprotein 1) | 0.86 | 180-196 | - | 1.000 | 0.877 | TtTAATgcg*att*ccggt CpG10 CpG9 |
| V$HOMF/HHEX.01Haematopoietically expressed homeobox, proline-rich homeodomain protein | 0.95 | 187-203 | - | 1.000 | 0.950 | Ggagggtt*tTAAT*gcga Cpg11 CPG10 |
| V$TBPF/MTATA.01Muscle TATA box | 0.84 | 188-204 | + | 1.000 | 0.842 | Cgc*atTAAA*accctccg CpG10 CpG11 |
| V$MOKF/MOK2.02Ribonucleoprotein-associated zinc finger protein MOK-2 (human) | 0.98 | 194-214 | + | 1.000 | 0.985 | Aaaaccctccgaa*CCTT*tggg CpG11 |
| V$NR2F/COUP.01Chicken ovalbumin upstream promoter1 (COUP-TFI) and chicken ovalbumin upstream promoter 2 (COUP-TFII), DR1 sites | 0.82 | 198-222 | - | 1.000 | 0.829 | Ccgcgct*g*cc*ca*a*AGGTt*cggaggg Cpg13/12 CpG11 |
| V$PAX5/PAX5.01B cell-specific activator protein | 0.79 | 207-235 | + | 0.952 | 0.802 | CctttgGG*CA*gc*g*cg*g*caccg*g*ggctcac  **CpG12/13 CpG14 CpG15** |
| V$HEAT/HSF2.01Heat shock factor 2 | 0.88 | 285-309 | + | 1.000 | 0.890 | Tcaggatagctcg*GAA*Ccc*t*ctgag CpG20 |
| V$EKLF/EKLF.01Erythroid krueppel-like factor (EKLF) | 0.89 | 294-310 | - | 1.000 | 0.925 | G*c*tcag*aGGGT*tccgag CpG20 |
